# Mechanistic understanding of metabolic cross-talk between *Aloe vera* and native soil bacteria for growth promotion and secondary metabolites accumulation

**DOI:** 10.3389/fpls.2025.1577521

**Published:** 2025-03-27

**Authors:** Neha Singh Chandel, H. B. Singh, Anukool Vaishnav

**Affiliations:** Department of Biotechnology, GLA University, Mathura, Uttar Pradesh, India

**Keywords:** *Aloe vera*, flavonoids, PGPR, plant-soil-microbe interaction, metabolic communication, secondary metabolites

## Abstract

Plants release a wealth of metabolites into the rhizosphere that can influence the composition and activity of microbial communities. These communities, in turn, can affect the growth and metabolism of the host plant. The connection between medicinal plant and its associated microbes has been suggested, yet the mechanisms underlying selection of indigenous microbes, and their biological function in medicinal plants are largely unknown. In this study, we investigated how the *Aloe vera* plants select its rhizosphere bacteria and examined their functional roles in relation to plant benefit. We utilized two native plant growth promoting rhizobacterial (PGPR) strains of *Aloe vera*: *Paenibacillus* sp. GLAU-BT2 and *Arthrobacter* sp. GLAU-BT16, as either single or consortium inoculants for plant growth experiment. We analyzed non-targeted root metabolites in the presence of both single and consortium bacterial inoculants and confirmed their exudation in the rhizosphere. The GC-MS analysis of metabolites revealed that the bacterial inoculation in *Aloe vera* plants amplified the abundance of flavonoids, terpenes and glucoside metabolites in the roots, which also exuded into the rhizosphere. Flavonoids were the most common prevalent metabolite group in individual and consortium inoculants, highlighting their role as key metabolites in interactions with rhizosphere microbes. In addition, the bacterial inoculants significantly increased antioxidant activity as well as total phenolic and flavonoid content in the leaves of *Aloe vera*. In conclusion, we propose a model of circular metabolic communication in which rhizosphere bacteria induce the production of flavonoids in plants. In turn, the plant releases some of these flavonoids into the rhizosphere to support the indigenous microbial community for its own benefit.

## Introduction

Soil microbes have undeniably established close association with their host plants for ages, ranging from mutualistic to parasitic. In mutualistic interactions, there are dynamic changes in the physiology and metabolism of the symbiotic partners (Singh et al., 2022). This relationship provides various benefits to the host plant, including growth promotion, improved nutrient uptake, enhanced tolerance against biotic and abiotic stresses, and modulation of metabolic pathway to accumulate more bioactive metabolite contents (Kumar and Nautiyal, 2022). In return, plants provide a nutrient-rich environment for the survival of selective microbes in their surroundings. The association of plant roots with numerous soil organisms is an interesting ecological niche known as the rhizosphere, which is beneficial for most plant symbionts (Maitra et al., 2024). Plants use a variety of mechanisms to modulate their microbiome, including the exudation of secondary metabolites and the coordinated action of different defense responses (Afridi et al., 2024). The relationships that exist between plants and rhizosphere microbes are dynamic and can be species-specific or environment-dependent (Berihu et al., 2023). Host plants can actively modulate the assembly of their rhizosphere microbiome in response to stressors and other environmental factors (Luo et al., 2023). Plants communicate with beneficial microorganisms in the rhizosphere through root exudates, creating a regulated microbial community. Changes in root microbiota throughout development influence soil-microbe feedback and rhizosphere chemistry, which are vital for survival in varying conditions (Korenblum et al., 2020). This dynamic response further emphasizes the need to understand the individual interactions between rhizosphere microbes and their hosts or specific crops, and the chemical basis of such interactions, which remains elusive in most cases and is a major reason for the failure of microbial products under field conditions (Bakker et al., 2018; Liu et al., 2019).

Plant growth-promoting rhizobacteria (PGPR) are beneficial bacteria found in the rhizosphere that form symbiotic relationships with plant roots through metabolic communication (Vaishnav et al., 2017; Singh et al., 2021; Singh et al., 2021). During this interaction, plants release signaling compounds, such as organic acids and phenolic compounds, to attract PGPR in the rhizosphere (Strehmel et al., 2014; Miller et al., 2019). Plants selectively exude these compounds based on their specific needs, which influences the composition of PGPR populations (Mashabela et al., 2022). In return, PGPR produce various traits that promote plant growth, including siderophores, organic acids for nutrient solubilization, and phytohormones (Vaishnav et al., 2014; Jain et al., 2014). These contributions lead to root elongation, improved overall growth, enhanced nutrient uptake, and increased plant defenses (Noumavo et al., 2013; Prasad et al., 2019). Among these beneficial bacteria, *Paenibacillus* spp. is the most abundant operational taxonomic unit (OTU) in the plant microbiome (Langendries and Goormachtig, 2021). It promotes growth through nitrogen fixation, phosphate solubilization, and phytohormone production, while also offering protection against pests and pathogens through antimicrobial compounds (Grady et al., 2016). Inoculating soil with *P. polymyxa* has been shown to improve microbial diversity and crop yields. For instance, in poplar plantations, it increased beneficial bacteria and reduced harmful fungi (Sui et al., 2019). Similarly, *Arthrobacter* spp. also hold significant potential as PGPR and can be found in various extreme environments, such as saline areas, drought-prone regions, and polluted soils. They play a vital role in protecting plants from abiotic stresses and enhancing plant nutrition, health, and yield (Sziderics et al., 2007; Tiwari et al., 2011; Qin et al., 2014). As key PGPR, *Arthrobacter* spp. improve iron acquisition by reducing and dissolving Fe^3+^ in the soil and also promote the growth of both leguminous and monocot plants by producing beneficial volatile compounds (Velázquez-Becerra et al., 2011; del-Carmen-Orozco-Mosqueda et al., 2013; Flores-Cortez et al., 2019).

In recent years, several studies on medicinal plants have enhanced our understanding of how the plant symbionts impact the quality of the host plant by influencing their medicinal metabolite compounds (Sharma et al., 2023). The microbial symbionts associated with medicinal plants have shown the ability to produce new leads of secondary metabolites with industrial and biotechnological implications, as well as stimulate plant growth and development (Wu et al., 2021). *Aloe vera* is highly sought after for its medicinal and cosmetic uses globally, attributed to therapeutic properties of its several secondary metabolites found in the leaf gel (Taher et al., 2024). Despite India being the largest producer of *Aloe vera*, it still struggles to meet the increasing demand for it (Lawal et al., 2021). Reproductive challenges and susceptibility to diseases pose threats to the productivity and yield of metabolite contents in *Aloe vera* (Kiran et al., 2017; Berhe et al., 2023). In addition, the excessive or unsustainable use of different cultivars of *Aloe vera* has placed them at a high risk of extinction, categorized in the red list by the International Union for Conservation of Nature (IUCN) (Bachman et al., 2020). Although efforts have been made to increase productivity and yield of metabolite contents in *Aloe vera*, they have limitations in large-scale production due to limited knowledge of the defined metabolic pathway in *Aloe vera*. The existing approaches such as heterologous gene expression, plant cell culture engineering, and breeding methods are insufficient to meet industrial demand (Isah et al., 2018; Wu et al., 2021). Reports indicate that the production of bioactive secondary metabolites in medicinal plants is stimulated by their associated microbes (Wu et al., 2021; Tripathi et al., 2022). The isolation of these plant associated microbes could be highly beneficial for large-scale production of bioactive secondary metabolites with medicinal value. However, the understanding of the relationship between the accumulation of bioactive components of *Aloe vera* and the root-associated microbiome is still limited and fragmented in the few studies (Chandra et al., 2024).

In this study, we aimed to assess how *Aloe vera* recruits beneficial bacteria in the rhizosphere. To do this, we used two native PGPR strains *Paenibacillus* sp. GLAU-BT2 (GenBank accession number- PV083208) and *Arthrobacter* sp. GLAU-BT16 (GenBank accession number- PV083209), previously isolated from *Aloe vera*’s rhizosphere (Unpublished data). These bacterial strains were used as a single and consortium inoculant to perform plant growth experiment with *Aloe vera*. We then assessed the effects of these bacterial strains on root metabolites and their secretion in the rhizosphere soil as well. We further investigated the bacterial effects on plant growth and secondary metabolites accumulation in the leaves of *Aloe vera*.

## Materials and methods

### Biological materials

We utilized pure cultures of two PGPR isolates *Paenibacillus* sp. GLAU-BT2 and *Arthrobacter* sp. GLAU-BT16, previously isolated from *Aloe vera* rhizosphere and stored in slant cultures at 4°C in the Plant biotechnology laboratory, Department of Biotechnology, GLA University, Mathura, India. *Aloe vera’*s root suckers were collected from a field crop growing in Horticulture Garden, GLA University, Mathura, India. All root suckers were surface sterilized before using in pot experiment. For bacterial inoculum preparation, a 24-hour-old bacterial culture was used, and the cell pellet was resuspended in sterile water to achieve a final concentration of 10^8^ CFU/mL for inoculation.

### Cross-compatibility of bacterial strains and development of the consortium inoculum

The cross-compatibility of both *Paenibacillus* sp. GLAU-BT2 and *Arthrobacter* sp. GLAU-BT16 was tested for growth by spotting them on Luria-Bertani (LB) agar plates and for biofilm formation in microtiter plates under broth medium. Bacterial growth occurring together indicated that the selected strains can work together as a consortium. The consortium was formed by growing the bacterial strains individually in LB broth until reaching the desired population (10^8^ CFU). After that, both cultures were mixed in equal ratios and used to inoculate *Aloe vera* rhizomes.

### Plant growth experiment with bacterial inoculation

The pot experiment aimed to assess the efficacy of both bacterial strains individually and in a consortium on the growth and metabolite contents of *Aloe vera* plants. This experiment was conducted in 5-liter earthen pots filled with sterilized soil. The bacterial inoculums were applied through dipping pre-sterilized root suckers of *Aloe vera* in the bacterial solutions for 1 hour before planting them in pots. This experiment was consisted of four treatment groups: (T1)- Control (without bacterial inoculation); (T2) GLAU-BT2 inoculation; (T3) GLAU-BT16 inoculation; (T4) Consortium inoculation (GLAU-BT2+GLAU-BT16). Each treatment was conducted with four replicates. The plants were watered weekly with sterile water, and all necessary plant protection measures were followed throughout the experiment. Plants were harvested 90 days after inoculation, and leaf parameters were measured, including number of leaves and their width and fresh weight.

### Biochemical estimation of secondary metabolites in the leaves of *Aloe vera*

After harvesting, the plants were taken for testing phytochemical estimations. The plant leaves were dried in the oven at 50 °C for 48 hours, ground into powder, and used to estimate the total phenol, flavonol, flavonoid contents, and DPPH, following the protocol provided by Sharma et al. (2014). Total phenol contents were measured using Folin-Ciocalteu reagent and absorbance was taken at 765 nm. The total phenolic contents were expressed in terms of gallic acid equivalent (mg/g dry weight). Similarly, the total flavonoid contents were measured by aluminum chloride colorimetric method, and absorbance was measured at 415 nm. The total flavonoid contents were expressed in terms of quercetin equivalent (mg/g dry weight). Furthermore, the total flavonol contents were measured at an absorbance of 440 nm and expressed in terms of quercetin equivalent (mg/g dry weight). Additionally, the DPPH was used to measure the free radical scavenging activity of the leaf extract, and absorbance was measured by a spectrophotometer at 517 nm, in which ascorbic acid was used as the standard (Sharma et al., 2014). The antioxidant activity percentage (%) was determined by the following formula, whereas AC = absorbance of DPPH solution without extract and AE = absorbance of the tested extract.


Antioxidant activity ( %)=[(AC−AE)∕AC]×100

### Extraction of metabolites from root and rhizosphere soil

To verify the exudation of root metabolites in the rhizosphere, nontargeted metabolites were extracted from both the roots and the adhering soil. For the root extracts, 50 mg of dried root samples were ground, and metabolites were extracted using 1 mL of precooled methanol. The mixture was subjected for sonication on ice bath followed by centrifugation at 14,000 g for 20 minutes at 4°C. The supernatant was collected and concentrated to dryness under vacuum and finally dissolved in 400 µL of cold methanol for gas chromatography-mass spectrometry (GC-MS) analysis (Kang et al., 2019). For rhizosphere soil sampling, soil was collected at 1 cm from the roots by gently scraping the surface. Additionally, soil attached directly to the roots was shaken off and collected. The extraction of exudates from soil samples was done according to method of Eze and Amuji (2024) and was subsequently dissolved in methanol for final analysis. Two different reference controls were used in this experiment to eliminate native soil metabolites and bacterial metabolites without presence of plant. These controls involved setups with no plants grown in the pots: (A) only sterile soil in the pot and (B) soil inoculated with a consortium of microorganisms.

### Characterization of metabolite compounds through GC–MS analysis

The purified methanolic extract was subjected to GC-MS analysis in a Perkin Elmer GC-MS Clarus^®^ SQ 8 equipped with DB-5MS (Agilent, USA) capillary standard non-polar column with dimensions 0.25 mm OD x 0.25 µm ID x 30 m length. The instrument was set to an initial temperature of 40°C, and the injection port temperature was ensured at 220°C, interface temperature set 250°C, source kept at 220°C, oven temperature programmed as 75°C for 2 min, 150°C @ 10°C/min, up to 250°C at 10°C per min. The GC conditions were: 1:12 split, helium carrier at 20 psi. The MS conditions were positive ion mode, electron impact spectra at 70 eV. The mass spectral scan range was set at 50 to 600 Da. The MS peaks were determined by their scatter pattern. The linear regression coefficient was used to calculate the concentrations in the samples from peak areas obtained in the chromatographs. The bioactive molecules were identified by comparison of mass spectra with NIST 08 Mass Spectra Library (National Institute of Standards and Technology). The name, molecular weight, and structure were ascertained from NIST, PubChem, and HMDB databases (Leylaie and Zafari, 2018).

### Data processing and statistical analysis

Conformity with the assumptions of analysis of variance (ANOVA) were checked for the different data by ShapiroWilk test for normality and Levene’s test for homogeneity of variances using the “car” package in R 4.2.2 (R Core Team, 2022). The data were analyzed by one-way ANOVA, followed by *post hoc* Tukey’s test to separate treatment means if ANOVA results were significant (*p*<0.05). For comparative analysis of metabolites in different treatments, the GC-MS data from six distinct groups were imported into separate data frames using RStudio. The initial step involved processing the data to generate chord diagrams that illustrate the connections between compounds and their respective properties. To achieve this, we created new data frames for each group, focusing on the “Compound” and “Properties” columns. Properties listed in a single cell and separated by commas were then expanded so that each cell contained only a single property, thereby ensuring the singularity of the “Properties” column. The information about compounds was replicated for the new rows to maintain consistency, reducing redundancy where the same property was previously recorded in multiple cells. Preprocessing included the removal of leading and trailing whitespaces from both columns. We used the circlize package (Gu et al., 2014) to create chord diagrams for each group, which were subsequently combined into a composite collage. For visualization of intersections among the sets of compounds, we utilized the UpSetR package (Conway et al., 2017), opting for this approach over a Venn diagram due to the complexity of having more than five sets, which can obscure intersection details in Venn diagrams. Color schemes were adjusted discretely to clearly differentiate between intersections and other visual representations.

## Results

### Co-inoculation of bacterial strains promote leaf growth parameters

**Figure 1:**
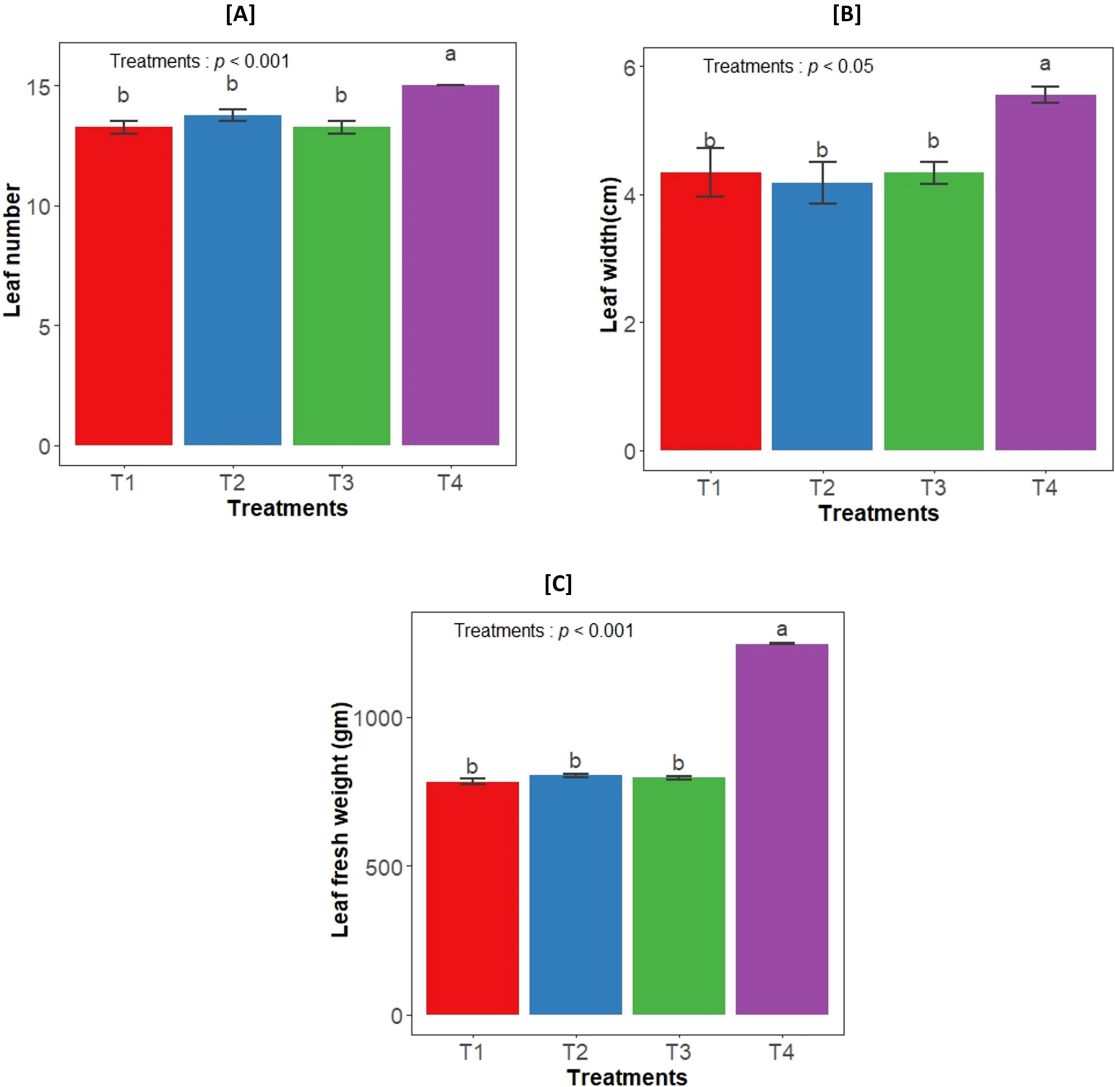
Effect of bacterial inoculation on **(A)** Leaf number, **(B)** Leaf width, and **(C)** Leaf fresh weight. T1- uninoculated plant (control); T2- GLAU-BT2 inoculation; T3- GLAU-BT16 inoculation; and T4- Consortium inoculation (GLAU-BT2 + GLAU-BT16). The results are presented as mean values± SE, (n=4). The presence of different letters on each bar indicates a statistically significant difference between the treatment means. The means were separated by Tukey’s *post-hoc* test (p<0.05), following a significant one-way ANOVA analysis.

The impact of both bacterial strains on the growth of *Aloe vera* plants was examined by determining different leaf growth parameters. There was a significant effect of different treatments in the leaf number (*F_3,12_* = 14.56, *p*<0.001), leaf width (*F_3,12_* = 5.5, *p*<0.05) and leaf fresh weight (*F_3,12_* = 1322, *p*<0.001). The highest value of leaf growth parameters were observed in consortium inoculation, while individual inoculation showed similar trend to control plants (**Figure 1**).

### Bacterial inoculation induces production of secondary metabolites in leaves

**Figure 2:**
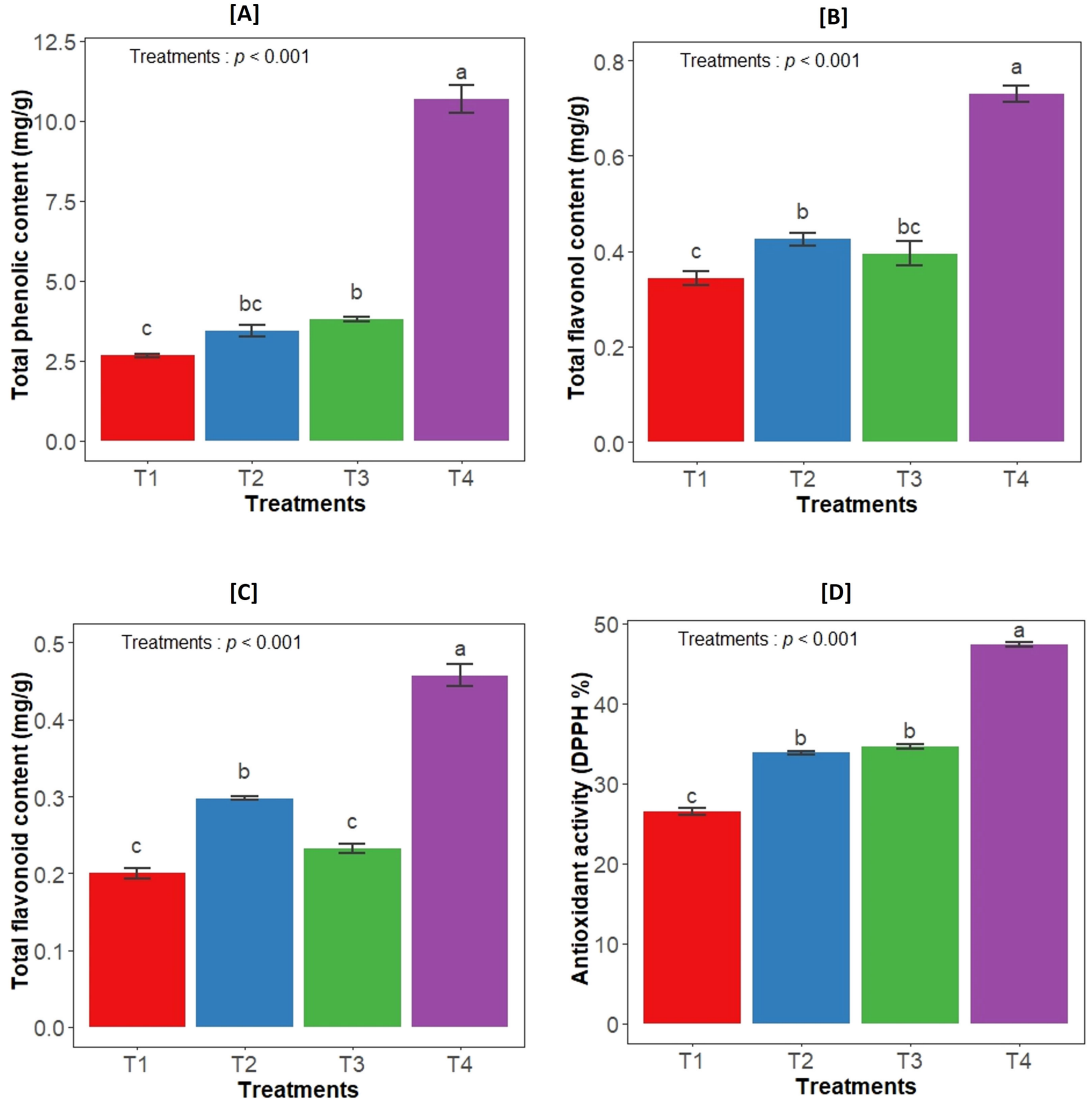
Effect of bacterial inoculation on **(A)** total phenolic content, **(B)** total flavonol content, **(C)** total flavonoid content and **(D)** antioxidant activity (DPPH). T1- uninoculated plant (control); T2- GLAU-BT2 inoculation; T3- GLAU-BT16 inoculation; and T4- Consortium inoculation (GLAU-BT2 + GLAU-BT16). The results are presented as mean values± SE, (n=4). The presence of different letters on each bar indicates a statistically significant difference between the treatment means. The means were separated by Tukey’s *post-hoc* test (p<0.05), following a significant one-way ANOVA analysis.

Different treatments had a significant impact on the total flavonoid content (*F_3,12_* = 187.7, *p*<0.001), total flavonol content (*F_3,12_* = 93.6, *p*<0.001) and total phenolic content (*F_3,12_* = 241.5, *p*<0.001) in the leaves of *Aloe vera* plant. The highest concentrations of all secondary metabolites were observed in consortium inoculated plants. In terms of total phenolic and flavonol content, both individual bacterial inoculations also resulted in significantly higher levels compared to control plants. However, for total flavonoids, the GLAU-BT16 individual inoculum showed a similar content to the control (**Figures 2A–C**). Additionally, the antioxidant activity measured by the DPPH assay in the leaf extract was significantly influenced by treatment groups (*F_3,12_* = 660.6, *p*<0.001). The highest DPPH activity was observed in the consortium treatment compared to the other treated plants (**Figure 2D**).

### Bacterial inoculated roots release distinctive metabolites into the rhizosphere

GC-MS profiling of metabolites from both roots and rhizosphere soil was conducted across all treatment groups, resulting in a final list of compounds after excluding their respective control groups. The number of compounds varied among the treatments. The highest number, 39 compounds, was observed in the rhizosphere soil of GLAU-BT16, while the lowest number, 35 compounds, was observed in the rhizosphere soil of GLAU-BT2 and in the roots of consortium inoculated plants. The final list of compounds, along with their properties, is summarized in **Supplementary Tables S1**-**S6**. Most compounds were detected within a retention time (RT) range of 3.1 to 31.3 minutes.

A total of 10 compounds were consistently identified in both the roots and rhizosphere soils across all treatment groups, indicating their stable presence. These compounds are “2,5-di-tert-Butylaniline”, “7-Tetradecyne”, “3-Octadecyne”, “trans-2-Decen-1-ol, methyl ether”, “1-Methylbicycloctane”, “Octadecadienoic acid, methyl ester”, “2H-Benzocyclohepten-2-one”, “Octadecatrienoic acid”, “3,4-Dimethyl-1-dimethyl(trimethylsilylmethyl)” and “Cyclooctene, 5,6-diethenyl”. Each treatment group also exhibited unique compounds that were not found in other groups. The highest number of unique compounds, 9, was identified in the rhizosphere soil of GLAU-BT16 inoculated treatment, which were absent in all other treatments.

In terms of root metabolite exudation into the rhizosphere, two unique compounds “2-Butenoic acid, 2-methyl” and “Pentanedioic acid, bis-dodecylamide” were exuded from the consortium treated roots into their rhizosphere soil. Additionally, two unique compounds, “1,3-dioxane-5,5-dimethanol, 2-hexyl” and “Cyclohexane, [6-cyclopentyl-3-(3-cyclopentylpropyl)hexyl]”, were exuded from the roots inoculated with GLAU-BT16, while one unique compound “Myricitrin” was exuded from the roots of GLAU-BT2 inoculated plants.

**Figure 3:**
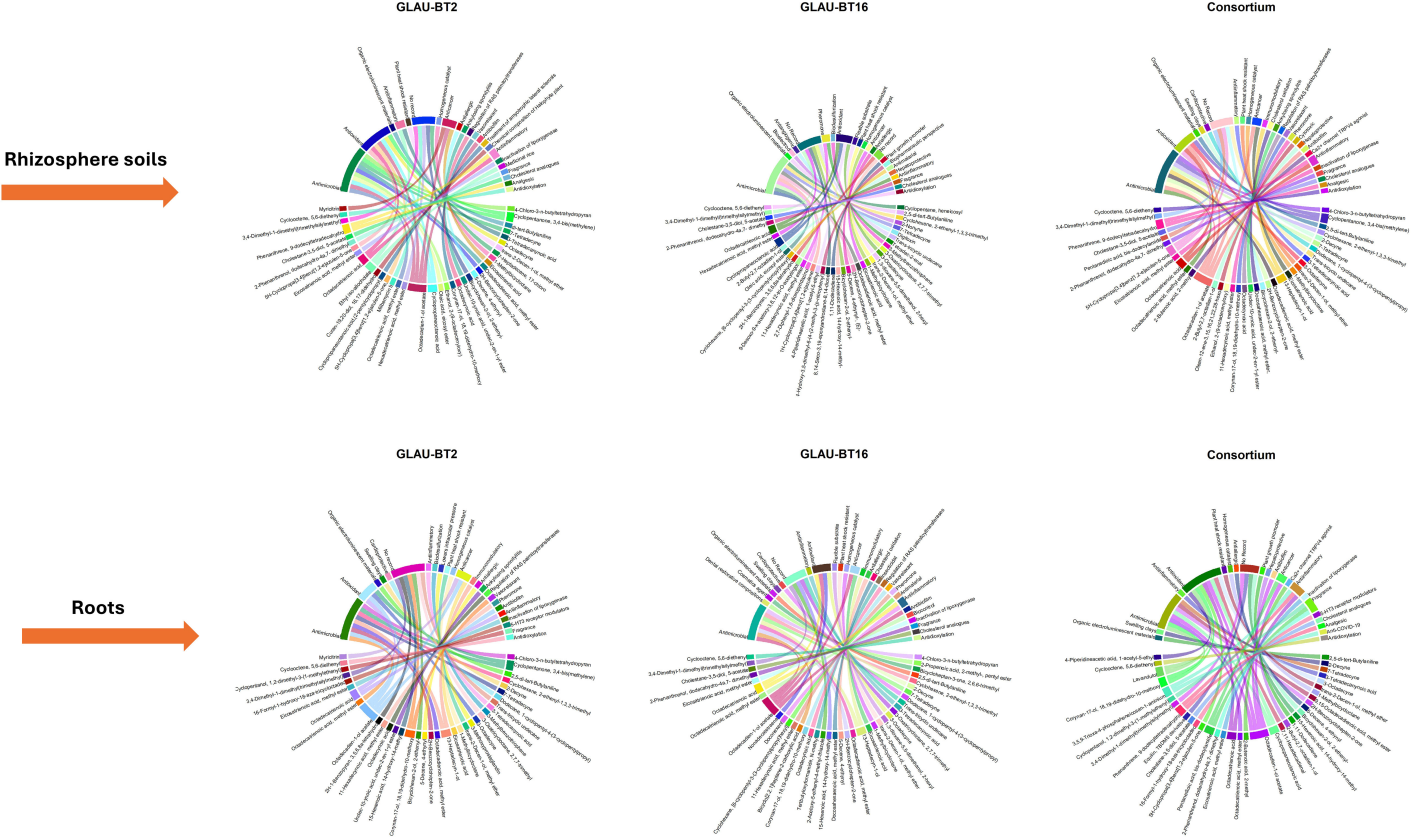
Chord diagrams illustrating the associations between compounds and properties across root metabolites and rhizosphere soil metabolites present in all bacterial treatments. The figure is presented as a collage, with each chord diagram representing a separate group. The diagrams are organized into with each plot titled according to its respective group.

**Figure 4:**
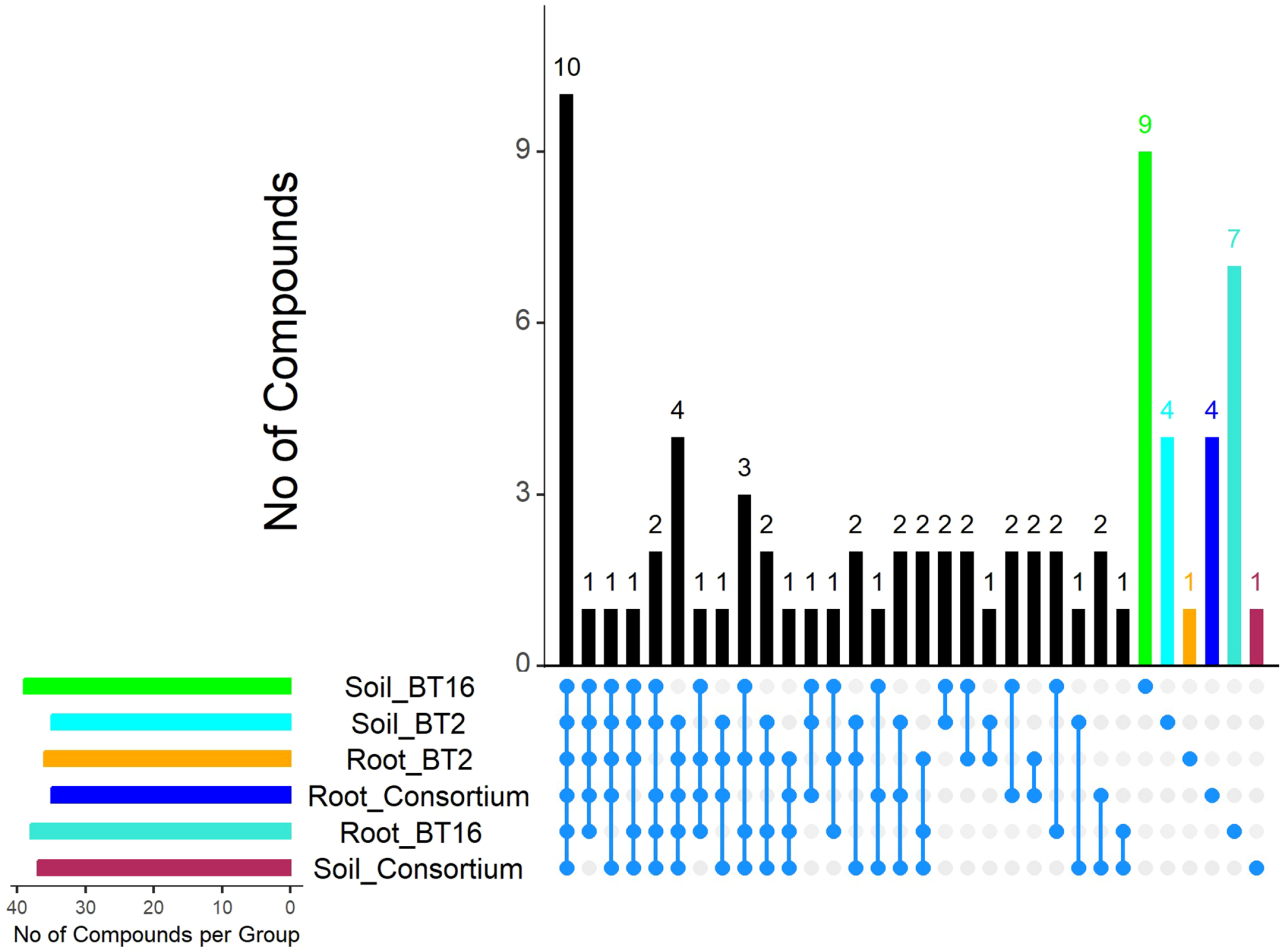
UpSet plot depicting the intersection statistics of compounds across root metabolites and rhizosphere soil metabolites present in all bacterial treatments. The bars are arranged in descending order of the number of intersections across the groups. The last six bars represent distinct values specific to each group. The colors of these bars are matched to the colors of the horizontal bars in the lower left corner, which indicate the total number of compounds in each group.

All identified compounds were researched in the available literature, and their reported activities are listed in the compiled **Supplementary Tables S1**-**S6**. The property “Antimicrobial” emerged as the most prevalent across all treatment groups, followed by “Antioxidant”. A collage of chord diagrams was created to visually represent the connections between individual compounds and their properties (**Figure 3**). An UpSet plot was also generated to understand the intersections of compounds among different treatment groups (**Figure 4**).

## Discussion

Plant-microbe interactions are complex and play a significant role in the metabolism of both plants and microbes. Using a metabolomics approach, researchers can identify low molecular weight metabolites involved in these interactions. Changes in these metabolites can provide insights into how plants and microbes respond to each other during specific physiological periods (Fiehn, 2002; Yang et al., 2017). Research on soil metabolomics has focused on understanding the relationships among plants, soil, and microbes by exploring “community metabolomics”, which considers both plant root exudates and microbial products (Jones et al., 2014; Huang et al., 2014). In this study, we examined the root metabolites of *Aloe vera* and their exudation into the rhizosphere while inoculating with two PGPR strains. Although collecting exudates from *Aloe vera* is challenging due to the large size of its roots, we aimed to identify common compounds present in both the roots and rhizosphere soil, even considering potential changes caused by microbial activity.

Our findings indicate that the consortium inoculum of *Paenibacillus* sp. GLAU-BT2 and *Arthrobacter* sp. GLAU-BT16 has a greater positive effect on the growth of *Aloe vera* plant than individual inoculants. Previous studies have demonstrated that using a mixed inoculant, rather than individual, promotes plant growth by increasing the number of surviving cells in their natural environments (Tittabutr et al., 2007; Sharma et al., 2017; Thanni et al., 2024). Our results align with previous studies on *Paenibacillus* sp. and *Arthrobacter* sp., which showed that plants inoculated with these bacterial strains exhibited enhanced growth parameters (Mohd Din et al., 2020; Chhetri et al., 2022; Jia et al., 2022; Özdoğan et al., 2022). Notably, co-inoculating *A. ureafaciens* DnL1-1 and *Trichoderma harzianum* significantly boosts biomass in wheat (Yang et al., 2021). In our results, the higher leaf biomass observed in bacterial-inoculated plants suggest that PGPR strains may increase nutrient uptake in *Aloe vera* plants through their nutrient solubilizing activities, as seen in GLAU-BT2 and GLAU-BT16 strains (Unpublished data). Earlier research also highlights nutrient solubilizing and other plant growth promoting properties in various strains of *Paenibacillus* and *Arthrobacter* (Banerjee et al., 2010; Grady et al., 2016; Chhetri et al., 2022).

**Figure 5:**
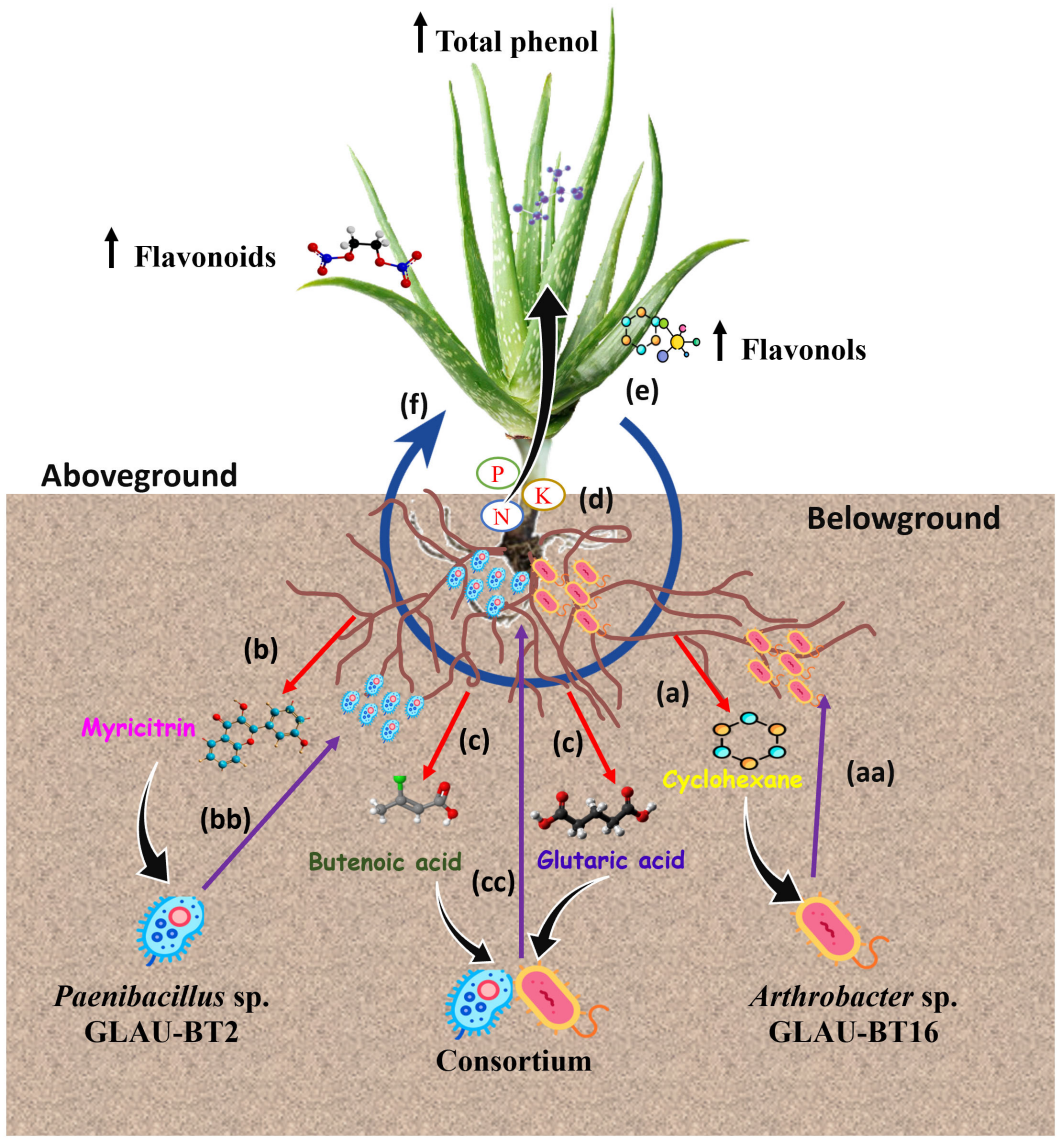
A model framework illustrating circular metabolic communication within the Aloe vera rhizosphere. Our findings suggest the following steps in metabolic communication: **(a)** Roots exude ‘Cyclohexane’ in response to *Arthrobacter* sp. GLAU-BT16. **(aa)** In response to ‘Cyclohexane’, *Arthrobacter* sp. migrate towards the rhizosphere, increasing their abundance. **(b)** Roots exude ‘Myricitrin’ in response to *Paenibacillus* sp. GLAU-BT2. **(bb)** In response to ‘Myricitrin’, *Paenibacillus* sp. move towards the rhizosphere and increase their abundance. **(c)** In response to consortium inoculation of GLAU-BT2 and GLAU-BT16, roots exude ‘Butanoic acid’ and ‘Glutaric acid’. **(cc)** Both *Paenibacillus* sp. GLAU-BT2 and *Arthrobacter* sp. GLAU-BT16 move towards the rhizosphere, increasing their populations in response to ‘Butanoic acid’ and ‘Glutaric acid’. **(d)** In the rhizosphere, these bacteria (GLAU-BT2 and GLAU-BT16) solubilize nutrients, aiding in their uptake by plants. **(e)** The enhanced nutrient uptake leads to an increase in the accumulation of total phenols, flavonoids, and flavonol in the plants. **(f)** Some of the accumulated metabolites are recirculated in the rhizosphere.

The co-inoculation of GLAU-BT2 and GLAU-BT16 in the host plant *Aloe vera* significantly increased the leaf metabolite contents, including total flavonoids, flavonol, phenolic compounds, and total antioxidant activity. The therapeutic benefits of *Aloe vera* are attributed to the antioxidant properties of its phytochemical components, which can scavenge free radicals and reduce oxidative damage associated with various plant diseases (Mahapatra, 2021; Singh and Vaishnav, 2022; Nwozo et al., 2023). These findings align with previous studies that demonstrated that plants inoculated with different species of *Arthrobacter* induced the accumulation of secondary metabolites, particularly influencing carbohydrate metabolism in the plant leaves (Ramírez-Ordorica et al., 2020; Chhetri et al., 2022). Similarly, a co-inoculation experiment with *Paenibacillus* and *Bacillus subtilis* in wheat cultivars revealed a differential accumulation of compounds across several classes of metabolites, including phenylpropanoids, organic acids, lipids, organoheterocyclic compounds, and benzenoids in leaf tissue (Mashabela et al., 2022). The higher accumulation of flavonoids and phenolic compounds is primarily exuded from the roots to attract beneficial microbes in the rhizosphere (Gu et al., 2020; Sharma et al., 2021; Korenblum et al., 2022). In our findings, we also observed flavonoid compound ‘Myricitrin’ especially in *Paenibacillus* sp. GLAU-BT2 treated plant roots as well as in rhizosphere soil. It can be suggested that the presence of *Paenibacillus* sp. GLAU-BT2 and *Arthrobacter* sp. GLAU-BT16 in the *Aloe vera* rhizosphere may result from the exudation of flavonoid and phenolic compounds (Reynolds, 1985). Once these microbes colonize the rhizosphere, they stimulate nutrient uptake in the host plant, leading to increased production of flavonoids and phenolic compounds. These compounds are then exuded and recirculated in the rhizosphere, allowing beneficial microbes to enhance their population and colonization within the host plant (**Figure 5**). This hypothesis is based on the concept of a circular metabolic economy in plant-microbe interactions, where flavonoids, the most studied class of chemical exudates, have diverse effects on soil microorganisms (Korenblum et al., 2022).

The GC-MS analysis identified various categories of compounds in both root and rhizosphere soil. Plant root exudates play a significant role in transforming and modifying the conditions of the rhizosphere. These exudates are often considered the first line of communication between plants and the microorganisms residing in the rhizosphere (Oburger et al., 2009; Sharma et al., 2021). The presence of antimicrobial metabolites in both the root and rhizosphere suggests that these compounds inhibit the growth of pathogenic microbes in the soil and reduce competition for *Paenibacillus* sp. GLAU-BT2 and *Arthrobacter* sp. GLAU-BT16, thus facilitating their growth in the rhizosphere. Moreover, *Arthrobacter* spp. have been reported to produce antimicrobial metabolites that exhibit biocontrol activity; for instance, *Arthrobacter agilis* produces dimethylhexadecylamine, which inhibits the growth of phytopathogenic fungi *in vitro* (Velázquez-Becerra et al., 2013). Additionally, interactions between wheat and *A. ureafaciens* DnL1-1, as well as *Trichoderma harzianum*, led to the modulation of soil metabolites, particularly amino acids, organic acids, triterpenoids, coumarins, and flavonoid contents in the rhizosphere soil (Yang et al., 2021).

Benzenoids and their derivatives were the most detected compounds in the metabolites of both root and rhizosphere soil, followed by lipids and organoheterocyclic compounds. These specialized aromatic metabolites, often classified as volatile organic compounds (VOCs), are produced by microorganisms and play crucial roles in plant defense, stress response, and interactions at the plant–microbe interface (Misztal et al., 2015; Vaishnav et al., 2017; Lackus et al., 2021). Their significance in belowground plant-plant and plant-microbe communications has gained increased attention (Schenkel et al., 2018; Singh et al., 2021). Furthermore, phenolic compounds serve as substrates or signaling molecules for various soil microbes (Badri et al., 2013). They enhance plant defenses against pathogens and contribute to abiotic stress responses (Irfan et al., 2019).

Other common compounds included organic acids and fatty acids (Butenoic acid, Hexenoic acid, 3-Tetradecynoic acid, Eicosatrienoic acid, Octadecynoic acid, and Piperidineacetic acid) were present in the metabolites of both root and rhizosphere soil from bacterial treatments. Organic acids are common exudates that have gained interest for their diverse roles as metabolites in the rhizosphere, as they can be produced by both plant roots and microorganisms (Tuason and Arocena, 2009). These acids facilitate nutrient solubilization, promote microbial growth, detoxify harmful and influence bacterial chemotaxis (Menezes-Blackburn et al., 2016; Jiang et al., 2017; Macias-Benitez et al., 2020). Additionally, fatty acids serve as energy reserves and are crucial for membrane lipids, acting as markers for microorganisms while also playing a role in plant defense (Kang et al., 2019).

Moreover, terpenes, fatty aldehydes, glucosides, and flavonoids were also detected in the metabolites of both root and rhizosphere soil. Terpenes, the largest group of plant secondary metabolites with vast diversity, have been found to have dual effects on soil microbes. Terpenes are reported to promote the proliferation of bacterial strains belonging to Proteobacteria while inhibiting the growth of Actinobacteria strains (Bai et al., 2021). Similarly, the biosynthesis of glucoside metabolites in plants is crucial for interaction with soil microbes and in shaping the rhizosphere community for their host plants (Hu et al., 2018; Koprivova et al., 2019). These findings emphasize the importance of understanding the role of root metabolites in the rhizosphere for attracting PGPR strains and enhancing the accumulation of secondary metabolites within plant tissue.

## Conclusion

Our observations provided insights into a proposed model of circular metabolic communication within the *Aloe vera* rhizosphere. In this model, *Aloe vera* plants may release flavonoids and phenolic metabolites that shape the rhizosphere bacteria. In turn, these rhizosphere bacteria produce phytohormones and nutrient-solubilizing enzymes in the soil, which enhance nutrient uptake by the plant roots. This process leads to improved plant growth and the accumulation of flavonoids, phenolic compounds, and other secondary metabolites, which then recirculate in the rhizosphere (**Figure 5**). Thus, this study enriches our understanding of the metabolic interactions between plants and microbes. This knowledge could facilitate the development of metabolome-engineering strategies aimed at enhancing plant growth, priming plants for defense, and promoting sustainable agriculture.

## Data Availability

The original contributions presented in the study are included in the article/**Supplementary Material**. Further inquiries can be directed to the corresponding author.
