# Supplementary material for: Mechanistic understanding of metabolic cross-talk between Aloe vera and native soil bacteria for growth promotion and secondary metabolites accumulation
Source: Front Plant Sci. 2025 Mar 27;16:1577521. doi: 10.3389/fpls.2025.1577521 (PMC11983424; doi:10.3389/fpls.2025.1577521)
Supplement: Supplementary file 1 [file Table1.docx]

**Mechanistic understanding of metabolic cross-talk between *Aloe vera* and native soil bacteria for growth promotion and secondary metabolites accumulation**

Neha Singh Chandel, H. B. Singh, Anukool Vaishnav*

Department of biotechnology, GLA University, Mathura-281122, Uttar Pradesh, India

*Corresponding author- Anukool Vaishnav; Email- [anukool.vaishnav@gla.ac.in](mailto:anukool.vaishnav@gla.ac.in); Mobile- +919983750974

**Table S1:** GC/MS profiling of metabolite compounds present in the **rhizosphere soil of consortium treated** plants and their properties as documented in available literature.

| **S. No.** | **Name of compound** | **Retention**  **time (RT)** | **Area%** | **Properties** | **References** |
| --- | --- | --- | --- | --- | --- |
| 1 | 4-Chloro-3-n-butyltetrahydropyran | 3,113 | 1,345 | Antimicrobial | Kassam et al., 2021 |
| 2 | Cyclopentanone, 3,4-bis(methylene) | 4,439 | 0,599 | Antioxidant, Antimicrobial | Mullaivendhan et al., 2024 |
| #3 | 2,5-di-tert-Butylaniline | 12,287 | 0,845 | Organic electroluminescent materials | Tsai et al., 2017 |
| 4 | Cyclohexene, 2-ethenyl-1,3,3-trimethyl | 12,852 | 0,482 | Antimicrobial | Li et al., 2012 |
| 5 | 2-Decyne | 14,113 | 0,565 | Swelling clays | Pinnavaia, 1987 |
| #6 | 7-Tetradecyne | 16,239 | 1,792 | Antimicrobial | El-Sheekh et al., 2020 |
| 7 | Dodecane, 1-cyclopentyl-4-(3-cyclopentylpropyl) | 16,354 | 0,478 | Cardioprotective | Varadharajan et al., 2016 |
| 8 | Trans-tricyclo undecane | 19,225 | 0,483 | No Record |  |
| 9 | 3-Tetradecanynoic acid | 19,32 | 1,077 | Antiinflammetory | Kolho et al., 2017 |
| #10 | 3-Octadecyne | 20,855 | 2,588 | Antioxidant | Gadhoumi et al., 2024 |
| #11 | trans-2-Decen-1-ol, methyl ether | 21,626 | 2,442 | Plant heat shock resistant | Liu et al., 2022 |
| #12 | 1-Methylbicycloctane | 22,326 | 0,86 | Homogeneous catalyst | Maurya, 2020 |
| 13 | Eicosatrienoic acid | 22,836 | 0,543 | Anticancer | Ediriweera et al., 2024 |
| 14 | 13-Heptadecyn-1-ol | 23,911 | 0,84 | Immunomodulatory, Antimicrobial | Ragunath and Ramasubramanian 2022 |
| #15 | Octadecadienoic acid, methyl ester | 24,262 | 1,393 | Antimicrobial | Nand et al., 2011 |
| #16 | 2H-Benzocyclohepten-2-one | 24,422 | 6,208 | Antimicrobial | AlAmery, 2020 |
| 17 | Bicyclohexan-2-ol, 2-ethenyl- | 24,877 | 1,627 | No Record |  |
| 18 | Docosahexaenoic acid, methyl ester | 24,972 | 0,626 | Cholesterol oxidation | Hu and Chen 2002 |
| 19 | Undec-10-ynoic acid, undec-2-en-1-yl ester | 25,762 | 0,607 | Ankylosing spondylitis | Winnett et al., 2017 |
| 20 | Octadecynoic acid | 26,012 | 1,221 | Regulation of RAS palmitoyltransferases | Yang et al., 2024 |
| 21 | Corynan-17-ol, 18,19-didehydro-10-methoxy | 26,062 | 0,773 | Vasorelaxant | Ahmad et al., 2010 |
| 22 | 11-Hexadecynoic acid, methyl ester | 27,888 | 0,664 | Pheromone | Hagström et al., 2013 |
| 23 | Ethanol, 2-(9-octadecenyloxy) | 28,313 | 0,651 | Antimicrobial | Mohy El-Din and Mohyeldin 2018 |
| *24 | Olean-12-ene-3,15,16,21,22,28-hexol | 28,958 | 0,945 | Cytotoxic | Fu et al., 2005 |
| 25 | 2-Butyl-2,7-octadien-1-ol | 29,053 | 2,303 | Hepatoprotective | Hota et al., 2022 |
| 26 | Octadecadien-1-ol acetate | 29,209 | 3,395 | Antimicrobial, Antibiofilm, Antioxidant, Anticancer | Rajput et al., 2021 |
| 27 | 2-Butenoic acid, 2-methyl | 29,264 | 1,004 | Ca2+ channel TRPV4 agonist | Alexander et al., 2013 |
| 28 | Octadecatrienoic acid, methyl ester | 29,614 | 0,738 | Antimicrobial | Ahmed and Othman, 2024 |
| #29 | Octadecatrienoic acid | 30,149 | 2,282 | Antioxidant, Antiinflammatory | Saha et al., 2012 |
| 30 | Eicosatrienoic acid, methyl ester | 30,229 | 2,196 | Inactivation of lipoxygenase | Kühn et al., 1991 |
| 31 | 5H-Cyclopropa[3,4]benz[1,2-e]azulen-5-one | 30,344 | 2,155 | No Record |  |
| 32 | 2-Phenanthrenol, dodecahydro-4a,7- dimethyl | 30,409 | 0,819 | Fragrance | Ishihara et al., 2005 |
| 33 | Pentanedioic acid, bis-dodecylamide | 30,489 | 1,443 | No Record |  |
| 34 | Cholestane-3,5-diol, 5-acetate | 30,679 | 2,233 | Cholesterol analogues | Nes, 2011 |
| 35 | Phenanthrene, 9-dodecyltetradecahydro | 30,744 | 1,256 | Analgesic, Antiinflammatory | Sayed et al., 2023 |
| #36 | 3,4-Dimethyl-1-dimethyl(trimethylsilylmethyl) | 30,949 | 1,507 | No Record |  |
| #37 | Cyclooctene, 5,6-diethenyl | 31,214 | 7,277 | Antidioxylation | Savoia et al., 2014 |

#The compounds highlighted in green were commonly found in all treatments.

*The compound highlighted in blue were only found in the rhizosphere soil inoculated with consortium treatment.

**Table S2:** GC/MS profiling of metabolite compounds present in the **roots of consortium treated plants** and their properties as documented in available literature.

| **S. No.** | **Name of compound** | **RT** | **Area%** | **Properties** | **References** |
| --- | --- | --- | --- | --- | --- |
| 1 | 2,5-di-tert-Butylaniline | 12,277 | 0,789 | Organic electroluminescent materials | Tsai et al., 2017 |
| 2 | 2-Decyne | 14,108 | 0,651 | Swelling clays | Pinnavaia, 1987 |
| 3 | 7-Tetradecyne | 18,35 | 1,056 | Antimicrobial | Sheekh et al., 2020 |
| 4 | 3-Tetradecanynoic acid | 19,31 | 0,79 | Antiinflammetory | Kolho et al., 2017 |
| 5 | 3-Octadecyne | 20,846 | 1,438 | Antioxidant | Gadhoumi et al., 2024 |
| 6 | trans-2-Decen-1-ol, methyl ether | 21,626 | 3,116 | Plant heat shock resistant | Liu et al., 2022 |
| 7 | 1-Methylbicycloctane | 22,331 | 0,666 | Homogeneous catalyst | Maurya, 2020 |
| 8 | Octadecadienoic acid, methyl ester | 24,252 | 0,913 | Antimicrobial | Nand et al., 2011 |
| 9 | 2H-Benzocyclohepten-2-one | 24,422 | 7,796 | Antimicrobial | AlAmery, 2020 |
| 10 | 5-Decene, 4-ethynyl | 24,767 | 0,662 | Antiallergic | Wang et al., 2001 |
| 11 | Bicyclohexan-2-ol, 2-ethenyl- | 24,882 | 2,951 | No Record |  |
| 12 | 15-Hexenoic acid, 14-hydroxy-14-methyl | 25,362 | 0,618 | No Record |  |
| 13 | 1,11-Dodecadiene | 25,998 | 0,966 | Plant growth promoter | Gamboa-Becerra et al., 2022 |
| 14 | 2-Butyl-2,7-octadien-1-ol | 29,004 | 1,245 | Hepatoprotective | Hota et al., 2022 |
| *15 | 7,11-Hexadecadienal | 29,039 | 0,69 | Antioxidant | Prabakaran et al., 2017 |
| 16 | Cyclopropaneoctanoic acid | 29,119 | 3,731 | Antimicrobial, Antioxidant | Chandrasekar et al., 2015 |
| 17 | Octadecadien-1-ol acetate | 29,214 | 0,994 | Antimicrobial, Antibiofilm, Antioxidant, Anticancer | Rajput et al., 2021 |
| #18 | 2-Butenoic acid, 2-methyl | 29,254 | 0,827 | Ca2+ channel TRPV4 agonist | Alexander et al., 2013 |
| 19 | Octadecatrienoic acid, methyl ester | 29,624 | 0,835 | Antimicrobial | Ahmed and Othman, 2024 |
| 20 | Octadecatrienoic acid | 30,199 | 0,794 | Antioxidant, Antiinflammatory | Saha et al., 2012 |
| 21 | Eicosatrienoic acid, methyl ester | 30,224 | 0,947 | Inactivation of lipoxygenase | Kühn et al., 1991 |
| 22 | 2-Phenanthrenol, dodecahydro-4a,7- dimethyl | 30,424 | 1,47 | Fragrance | Ishihara et al., 2005 |
| #23 | Pentanedioic acid, bis-dodecylamide (Glutaric acid) | 30,484 | 3,417 | Fragrance | Ishihara et al., 2005 |
| 24 | 5H-Cyclopropa[3,4]benz[1,2-e]azulen-5-one | 30,564 | 2,439 | Antioxidant | Barkatullah et al., 2024 |
| 25 | 16-Formyl-1-hydroxy-18-aza-tricycloctadec | 30,594 | 0,721 | 5-HT3 receptor modulators | Guzzo et al., 2015 |
| 26 | Cholestane-3,5-diol, 5-acetate | 30,624 | 0,765 | Cholesterol analogues | Nes, 2011 |
| *27 | Equilin, TBDMS derivative | 30,659 | 0,818 | No Record |  |
| 28 | Phenanthrene, 9-dodecyltetradecahydro | 30,704 | 3,219 | Analgesic, Antiinflammatory | Sayed et al., 2023 |
| 29 | 3,4-Dimethyl-1-dimethyl(trimethylsilylmethyl) | 30,96 | 3,647 | No Record |  |
| 30 | Cyclopentanol, 1,2-dimethyl-3-(1-methylethenyl) | 31,015 | 1,159 | Fragrance | Dong et al., 2015 |
| *31 | 3,5,9-Trioxa-4-phosphaheneicosan-1-aminium | 31,08 | 1,874 | Antiinflammatory, Anti-COVID-19 | Ratheesh et al., 2022 |
| 32 | Corynan-17-ol, 18,19-didehydro-10-methoxy | 31,13 | 1,341 | Antimicrobial, Antioxidant | Yahaya et al., 2021 |
| *33 | Lavandulol | 31,2 | 1,094 | Antimicrobial, Antioxidant | Özdemir et al., 2022 |
| 34 | Cyclooctene, 5,6-diethenyl | 31,245 | 1,587 | Antidioxylation | Savoia et al., 2014 |
| 35 | 4-Piperidineacetic acid, 1-acetyl-5-ethyl | 31,28 | 1,804 | Antioxidant | Udayaprakash et al., 2015 |

# Compounds highlighted in yellow were commonly present in the consortium treatment roots as well as in its rhizosphere soil.

*The compounds highlighted in blue were only found in the roots inoculated with consortium treatment.

**Table S3:** GC/MS profiling of metabolite compounds present in the **rhizosphere soil of GLAU-BT16** treated plants and their properties as documented in available literature.

| **S. No.** | **Name of compound** | **RT** | **Area%** | **Properties** | **References** |
| --- | --- | --- | --- | --- | --- |
| *1 | Cyclopentane, heneicosyl | 11,857 | 0,55 | Antimicrobial | Watanabe et al., 2003 |
| 2 | 2,5-di-tert-Butylaniline | 12,232 | 0,789 | Organic electroluminescent materials | Tsai et al., 2017 |
| 3 | Cyclohexene, 2-ethenyl-1,3,3-trimethyl | 12,822 | 0,537 | Antimicrobial | Li et al., 2012 |
| *4 | 2-Nonyne | 14,078 | 0,662 | Bioelectrod | Yoshitomi et al., 2021 |
| 5 | 7-Tetradecyne | 16,209 | 1,843 | Antimicrobial | El-Sheekh et al., 2020 |
| *6 | Digitoxin | 17,524 | 0,601 | Antiangiogenic | Boscaro et al., 2024 |
| 7 | Trans-tricyclo undecane | 19,2 | 2,136 | No Record |  |
| *8 | Tetradec-2-enal | 19,28 | 1,458 | Pheromone | Do et al., 2011 |
| 9 | 3-Methylbenzothiophene | 19,605 | 0,778 | Biodesulfurization | Parveen et al ., 2024 |
| 10 | 3-Oxatricycloctane, 2,7,7-trimethyl | 20,035 | 0,966 | No Record |  |
| 11 | 3-Octadecyne | 20,82 | 2,102 | Antioxidant | Gadhoumi et al., 2024 |
| 12 | 1,3-dioxane-5,5-dimethanol, 2-hexyl | 21,481 | 0,504 | Flexible substrate | Liang et al., 2015 |
| 13 | trans-2-Decen-1-ol, methyl ether | 21,616 | 9,032 | Plant heat shock resistant | Liu et al., 2022 |
| 14 | 1-Methylbicycloctane | 22,306 | 1,097 | Homogeneous catalyst | Maurya, 2020 |
| 15 | Eicosatrienoic acid | 22,826 | 0,523 | Anticancer | Ediriweera et al., 2024 |
| 16 | Octadecadienoic acid, methyl ester | 24,247 | 0,948 | Antimicrobial | Nand et al., 2011 |
| 17 | 2H-Benzocyclohepten-2-one | 24,407 | 3,433 | Antimicrobial | AlAmery, 2020 |
| 18 | 5-Decene, 4-ethynyl | 24,757 | 0,696 | Antiallergic | Wang et al., 2001 |
| 19 | Bicyclohexan-2-ol, 2-ethenyl- | 24,862 | 2,95 | No Record |  |
| 20 | 15-Hexenoic acid, 14-hydroxy-14-methyl | 25,327 | 0,828 | No record |  |
| 21 | 1,11-Dodecadiene | 25,987 | 1,596 | Plant growth promoter | Gamboa-Becerra et al., 2022 |
| *22 | 8,14-Seco-3,19-epoxyandrostane-8,14-dione | 27,578 | 0,8 | No Record |  |
| *23 | 4-Hydroxy-3,5-dimethyl-6-(4-(2-methyl-3-(p-nitrophenyl) | 27,633 | 0,798 | Biopharmaceutic perspective | Yang et al., 2024 |
| 24 | 4-Piperidineacetic acid, 1-acetyl-5-ethyl | 27,693 | 0,66 | Antioxidant | Udayaprakash et al., 2015 |
| *25 | 1H-Cyclopropa[3,4]benz[1,2-e]azulene | 27,713 | 0,527 | Antimicrobial | Idan et al., 2015 |
| *26 | 2,7-Diphenyl-1,6-dioxopyridazino | 27,788 | 0,563 | Antimicrobial | Altameme et al., 2015 |
| 27 | 11-Hexadecynoic acid, methyl ester | 27,893 | 0,487 | Pheromone | Hagström et al., 2013 |
| *28 | 9-Desoxo-9-x-acetoxy-3,8,12-tri-O-acetylingol | 27,918 | 0,615 | Antimicrobial | Shareef et al., 2016 |
| 29 | 2H-1-Benzopyran, 3,5,6,8a-tetrahydro | 28,068 | 1,211 | No record |  |
| 30 | Cyclohexane, [6-cyclopentyl-3-(3-cyclopentylpropyl)hexyl] | 28,288 | 1,479 | Antimalarial | Nabih and Astra 1976 |
| 31 | Oleic acid, eicosyl ester | 28,498 | 0,935 | Antimicrobial | Hashem et al., 2022 |
| 32 | 2-Butyl-2,7-octadien-1-ol | 29,069 | 0,494 | Hepatoprotective | Hota et al., 2022 |
| 33 | Cyclopropaneoctanoic acid | 29,199 | 2,692 | Antimicrobial, Antioxidant | Chandrasekar et al., 2015 |
| 34 | Hexadecatrienoic acid, methyl ester | 29,599 | 0,753 | No Record |  |
| 35 | Octadecatrienoic acid | 30,124 | 1,077 | Antioxidant, Antiinflammatory | Saha et al., 2012 |
| 36 | 2-Phenanthrenol, dodecahydro-4a,7- dimethyl | 30,419 | 1,064 | Fragrance | Ishihara et al., 2005 |
| 37 | Cholestane-3,5-diol, 5-acetate | 30,629 | 1,178 | Cholesterol analogues | Nes, 2011 |
| 38 | 3,4-Dimethyl-1-dimethyl(trimethylsilylmethyl) | 30,924 | 4,037 | No Record |  |
| 39 | Cyclooctene, 5,6-diethenyl | 31,229 | 2,998 | Antidioxylation | Savoia et al., 2014 |

*The compounds highlighted in blue were only found in the rhizosphere soil inoculated with GLAU-BT16.

**Table S4:** GC/MS profiling of metabolite compounds present in **roots of GLAU-BT16 treated plants** and their properties as documented in available literature.

| **S. No.** | **Name of compound** | **RT** | **Area%** | **Properties** | **References** |
| --- | --- | --- | --- | --- | --- |
| 1 | 4-Chloro-3-n-butyltetrahydropyran | 3,118 | 0,59 | Antimicrobial | Kassam et al., 2021 |
| *2 | 2-Propenoic acid, 2-methyl-, pentyl ester | 5,399 | 0,604 | Dental restorative compositions | Stansbury et al., 2014 |
| *3 | Bicycloheptan-3-one, 2,6,6-trimethyl | 11,902 | 0,516 | Cosmetics agent | Nowak et al., 2022 |
| 4 | 2,5-di-tert-Butylaniline | 12,287 | 0,981 | Organic electroluminescent materials | Tsai et al., 2017 |
| 5 | Cyclohexene, 2-ethenyl-1,3,3-trimethyl | 12,862 | 0,622 | Antimicrobial | Li et al., 2012 |
| 6 | 2-Decyne | 14,123 | 0,765 | Swelling clays | Pinnavaia, 1987 |
| 7 | 7-Tetradecyne | 16,239 | 2,023 | Antimicrobial | El-Sheekh et al., 2020 |
| 8 | Dodecane, 1-cyclopentyl-4-(3-cyclopentylpropyl) | 16,374 | 0,551 | Cardioprotective | Varadharajan et al., 2016 |
| 9 | Trans-tricyclo undecane | 19,225 | 0,442 | No Record |  |
| 10 | 3-Tetradecanynoic acid | 19,315 | 0,607 | Antiinflammetory | Kolho et al., 2017 |
| 11 | 3-Oxatricycloctane, 2,7,7-trimethyl | 20,085 | 0,461 | No Record |  |
| 12 | 3-Octadecyne | 20,85 | 1,876 | Antioxidant | Gadhoumi et al., 2024 |
| #13 | 1,3-dioxane-5,5-dimethanol, 2-hexyl | 21,496 | 0,526 | Flexible substrate | Liang et al., 2015 |
| 14 | trans-2-Decen-1-ol, methyl ether | 21,636 | 4,002 | Plant heat shock resistant | Liu et al., 2022 |
| 15 | 1-Methylbicycloctane | 22,336 | 0,965 | Homogeneous catalyst | Maurya, 2020 |
| 16 | Eicosatrienoic acid | 22,841 | 0,6 | Anticancer | Ediriweera et al., 2024 |
| 17 | 13-Heptadecyn-1-ol | 23,907 | 0,536 | Immunomodulatory, Antimicrobial | Ragunath and Ramasubramanian 2022 |
| 18 | Octadecadienoic acid, methyl ester | 24,262 | 2,039 | Antimicrobial | Nand et al., 2011 |
| 19 | 2H-Benzocyclohepten-2-one | 24,427 | 16,802 | Antimicrobial | AlAmery, 2020 |
| 20 | 5-Decene, 4-ethynyl | 24,782 | 1,037 | Antiallergic | Wang et al., 2001 |
| 21 | Docosahexaenoic acid, methyl ester | 24,902 | 4,805 | Cholesterol oxidation | Hu and Chen 2002 |
| 22 | 15-Hexenoic acid, 14-hydroxy-14-methyl | 25,352 | 0,778 | No Record |  |
| *23 | 2-Acetoxy-5-ethenyl-4-methyl-thiazole | 25,447 | 0,686 | No Record |  |
| *24 | Tertbutyloxyformamide, N-methyl | 25,607 | 1,018 | Insecticidal | Hussein et al., 2016 |
| 25 | Octadecynoic acid | 26,007 | 1,474 | Regulation of RAS palmitoyltransferases | Yang et al., 2024 |
| 26 | Corynan-17-ol, 18,19-didehydro-10-methoxy | 26,082 | 0,458 | Vasorelaxant | Ahmad et al., 2010 |
| *27 | Bicyclo[2.2.1]heptane-2-carboxylic acid | 27,138 | 0,5 | Antimicrobial | Shibnev et al., 2017 |
| 28 | 11-Hexadecynoic acid, methyl ester | 27,893 | 0,633 | Pheromone | Hagström et al., 2013 |
| #29 | Cyclohexane, [6-cyclopentyl-3-(3-cyclopentylpropyl)hexyl] | 28,298 | 0,797 | Antimalarial | Nabih and Astra 1976 |
| *30 | Doconexent | 28,558 | 0,428 | Antiinflammatory | de Oliveira et al., 2024 |
| *31 | Nonadecatetraene | 28,648 | 0,956 | Antioxidant | Palani et al., 2011 |
| 32 | Octadecadien-1-ol acetate | 29,204 | 1,106 | Antimicrobial, Antibiofilm, Antioxidant, Anticancer | Rajput et al., 2021 |
| 33 | Octadecatrienoic acid | 30,139 | 0,813 | Antioxidant, Antiinflammatory | Saha et al., 2012 |
| 34 | Eicosatrienoic acid, methyl ester | 30,229 | 1,279 | Inactivation of lipoxygenase | Kühn et al., 1991 |
| 35 | 2-Phenanthrenol, dodecahydro-4a,7- dimethyl | 30,429 | 0,515 | Fragrance | Ishihara et al., 2005 |
| 36 | Cholestane-3,5-diol, 5-acetate | 30,694 | 12,609 | Cholesterol analogues | Nes, 2011 |
| 37 | 3,4-Dimethyl-1-dimethyl(trimethylsilylmethyl) | 30,944 | 1,381 | No Record |  |
| 38 | Cyclooctene, 5,6-diethenyl | 31,239 | 1,92 | Antidioxylation | Savoia et al., 2014 |

# Compounds highlighted in yellow were commonly present in the GLAU-BT16 inoculated treatment roots as well as in its rhizosphere soil.

*The compounds highlighted in blue were only found in the roots inoculated with GLAU-BT16.

**Table S5:** GC/MS profiling of metabolite compounds present in the **rhizosphere soil of GLAU-BT2 treated plants** and their properties as documented in available literature.

| **S. No.** | **Name of compound** | **RT** | **Area%** | **Properties** | **References** |
| --- | --- | --- | --- | --- | --- |
| 1 | 4-Chloro-3-n-butyltetrahydropyran | 3,113 | 0,795 | Antimicrobial | Kassam et al., 2021 |
| 2 | Cyclopentanone, 3,4-bis(methylene) | 4,444 | 0,588 | Antioxidant, Antimicrobial | Mullaivendhan et al., 2024 |
| 3 | 2,5-di-tert-Butylaniline | 12,282 | 0,563 | Organic electroluminescent materials | Tsai et al., 2017 |
| 4 | 7-Tetradecyne | 18,349 | 0,947 | Antimicrobial | Sheekh et al., 2020 |
| 5 | 3-Tetradecanynoic acid | 19,315 | 0,666 | Antiinflammetory | Kolho et al., 2017 |
| 6 | 3-Octadecyne | 20,85 | 2,187 | Antioxidant | Gadhoumi et al., 2024 |
| 7 | trans-2-Decen-1-ol, methyl ether | 21,641 | 3,701 | Plant heat shock resistant | Liu et al., 2022 |
| *8 | 7-Heptadecene, 17-chloro | 21,836 | 0,599 | No record |  |
| 9 | 1-Methylbicycloctane | 22,336 | 0,715 | Homogeneous catalyst | Maurya, 2020 |
| 10 | Eicosatrienoic acid | 22,831 | 0,61 | Anticancer | Ediriweera et al., 2024 |
| 11 | Octadecadienoic acid, methyl ester | 24,257 | 1,022 | Antimicrobial | Nand et al., 2011 |
| 12 | 2H-Benzocyclohepten-2-one | 24,432 | 4,206 | Antimicrobial | AlAmery, 2020 |
| 13 | 5-Decene, 4-ethynyl | 24,782 | 0,804 | Antiallergic | Wang et al., 2001 |
| 14 | Bicyclohexan-2-ol, 2-ethenyl- | 24,892 | 3,457 | No record |  |
| 15 | Undec-10-ynoic acid, undec-2-en-1-yl ester | 25,752 | 0,627 | Ankylosing spondylitis | Winnett et al., 2017 |
| 16 | Octadecynoic acid | 26,012 | 1,056 | Regulation of RAS palmitoyltransferases | Yang et al., 2024 |
| 17 | Corynan-17-ol, 18,19-didehydro-10-methoxy | 26,077 | 0,782 | Vasorelaxant | Ahmad et al., 2010 |
| 18 | Ethanol, 2-(9-octadecenyloxy) | 28,318 | 1,359 | Antimicrobial | Mohy El-Din and Mohyeldin 2018 |
| 19 | Oleic acid, eicosyl ester | 28,493 | 0,78 | Antimicrobial | Hashem et al., 2022 |
| 20 | Cyclopropaneoctanoic acid | 29,129 | 2,083 | Antimicrobial, Antioxidant | Chandrasekar et al., 2015 |
| 21 | Octadecadien-1-ol acetate | 29,219 | 2,038 | Antimicrobial, Antibiofilm, Antioxidant, Anticancer | Rajput et al., 2021 |
| 22 | Hexadecatrienoic acid, methyl ester | 29,564 | 0,843 | No record |  |
| 23 | Octadecatrienoic acid, methyl ester | 29,614 | 1,312 | Antimicrobial | Ahmed and Othman, 2024 |
| *24 | Milbemycin b | 29,704 | 0,789 | No record |  |
| 25 | 5H-Cyclopropa[3,4]benz[1,2-e]azulen-5-one | 29,734 | 2,366 | Antioxidant | Barkatullah et al., 2024 |
| *26 | Curan-19,20-diol, 16,17-didehydro | 29,874 | 0,706 | Chemical composition of Halophyte plant | Altameme, 2017 |
| *27 | Ethyl iso-allocholate | 29,934 | 3,495 | Antiinflammetory | Johnson et al., 2020 |
| 28 | Octadecatrienoic acid | 30,144 | 3,519 | Antioxidant, Antiinflammatory | Saha et al., 2012 |
| 29 | Eicosatrienoic acid, methyl ester | 30,219 | 1,417 | Inactivation of lipoxygenase | Kühn et al., 1991 |
| 30 | 2-Phenanthrenol, dodecahydro-4a,7- dimethyl | 30,409 | 2,611 | Fragrance | Ishihara et al., 2005 |
| 31 | Cholestane-3,5-diol, 5-acetate | 30,644 | 2,685 | Cholesterol analogues | Nes, 2011 |
| 32 | Phenanthrene, 9-dodecyltetradecahydro | 30,769 | 0,88 | Analgesic, Antiinflammatory | Sayed et al., 2023 |
| 33 | 3,4-Dimethyl-1-dimethyl(trimethylsilylmethyl) | 30,949 | 3,883 | No record |  |
| 34 | Cyclooctene, 5,6-diethenyl | 31,244 | 2,936 | Antidioxylation | Savoia et al., 2014 |
| 35 | Myricitrin | 31,334 | 0,631 | Anticancer | Singh et al., 2023 |

*The compounds highlighted in blue were only found in the rhizosphere soil inoculated with GLAU-BT2.

**Table S6:** GC/MS profiling of metabolite compounds present in the **roots of GLAU-BT2 treated plants** and their properties as documented in available literature.

| **S. No.** | **Name of compound** | **RT** | **Area%** | **Properties** | **References** |
| --- | --- | --- | --- | --- | --- |
| 1 | 4-Chloro-3-n-butyltetrahydropyran | 3,118 | 0,958 | Antimicrobial | Kassam et al., 2021 |
| 2 | Cyclopentanone, 3,4-bis(methylene) | 4,449 | 0,548 | Antioxidant, Antimicrobial | Mullaivendhan et al., 2024 |
| 3 | 2,5-di-tert-Butylaniline | 12,282 | 0,678 | Organic electroluminescent materials | Tsai et al., 2017 |
| 4 | Cyclohexene, 2-ethenyl-1,3,3-trimethyl | 12,857 | 0,503 | Antimicrobial | Li et al., 2012 |
| 5 | 2-Decyne | 14,113 | 0,546 | Swelling clays | Pinnavaia, 1987 |
| 6 | 7-Tetradecyne | 16,234 | 1,733 | Antimicrobial | El-Sheekh et al., 2020 |
| 7 | Dodecane, 1-cyclopentyl-4-(3-cyclopentylpropyl) | 16,369 | 0,461 | Cardioprotective | Varadharajan et al., 2016 |
| 8 | Trans-tricyclo undecane | 19,22 | 0,632 | No record |  |
| 9 | 3-Tetradecanynoic acid | 19,315 | 0,81 | Antiinflammetory | Kolho et al., 2017 |
| 10 | 3-Methylbenzothiophene | 19,635 | 0,481 | Biodesulfurization | Parveen et al ., 2024 |
| 11 | 3-Oxatricycloctane, 2,7,7-trimethyl | 20,08 | 0,501 | No record |  |
| 12 | 3-Octadecyne | 20,855 | 2,653 | Antioxidant | Gadhoumi et al., 2024 |
| *13 | 3-Methoxyprostaglandin | 21,511 | 0,512 | Lowers intraocular pressure | PL, 1989 |
| 14 | trans-2-Decen-1-ol, methyl ether | 21,631 | 4,346 | Plant heat shock resistant | Liu et al., 2022 |
| 15 | 1-Methylbicycloctane | 22,341 | 0,93 | Homogeneous catalyst | Maurya, 2020 |
| 16 | Eicosatrienoic acid | 22,836 | 0,93 | Anticancer | Ediriweera et al., 2024 |
| 17 | 13-Heptadecyn-1-ol | 23,922 | 0,766 | Immunomodulatory, Antimicrobial | Ragunath and Ramasubramanian 2022 |
| 18 | Octadecadienoic acid, methyl ester | 24,262 | 1,671 | Antimicrobial | Nand et al., 2011 |
| 19 | 2H-Benzocyclohepten-2-one | 24,427 | 5,332 | Antimicrobial | AlAmery, 2020 |
| 20 | 5-Decene, 4-ethynyl | 24,777 | 0,725 | Antiallergic | Wang et al., 2001 |
| 21 | Bicyclohexan-2-ol, 2-ethenyl- | 24,887 | 3,67 | No record |  |
| 22 | Corynan-17-ol, 18,19-didehydro-10-methoxy | 25,082 | 0,989 | No record |  |
| 23 | 15-Hexenoic acid, 14-hydroxy-14-methyl | 25,342 | 0,75 | No record |  |
| 24 | Undec-10-ynoic acid, undec-2-en-1-yl ester | 25,772 | 0,727 | Ankylosing spondylitis | Winnett et al., 2017 |
| 25 | Octadecynoic acid | 26,012 | 1,388 | Regulation of RAS palmitoyltransferases | Yang et al., 2024 |
| 26 | 11-Hexadecynoic acid, methyl ester | 27,898 | 0,668 | Pheromone | Hagström et al., 2013 |
| 27 | 2H-1-Benzopyran, 3,5,6,8a-tetrahydro | 28,088 | 0,61 | No record |  |
| 28 | Octadecadien-1-ol acetate | 29,209 | 0,61 | Antimicrobial, Antibiofilm, Antioxidant, Anticancer | Rajput et al., 2021 |
| 29 | Octadecatrienoic acid, methyl ester | 29,619 | 1,041 | Antimicrobial | Ahmed and Othman, 2024 |
| 30 | Octadecatrienoic acid | 30,139 | 1,178 | Antioxidant, Antiinflammatory | Saha et al., 2012 |
| 31 | Eicosatrienoic acid, methyl ester | 30,259 | 0,664 | Inactivation of lipoxygenase | Kühn et al., 1991 |
| 32 | 16-Formyl-1-hydroxy-18-aza-tricycloctadec | 30,574 | 0,521 | 5-HT3 receptor modulators | Guzzo et al., 2015 |
| 33 | 3,4-Dimethyl-1-dimethyl(trimethylsilylmethyl) | 30,954 | 4,207 | No record |  |
| 34 | Cyclopentanol, 1,2-dimethyl-3-(1-methylethenyl) | 31,044 | 1,636 | Fragrance | Dong et al., 2015 |
| 35 | Cyclooctene, 5,6-diethenyl | 31,209 | 4,211 | Antidioxylation | Savoia et al., 2014 |
| #36 | Myricitrin | 31,309 | 2,262 | Anticancer | Singh et al., 2023 |

# Compound highlighted in yellow was commonly present in the GLAU-BT2 inoculated treatment roots as well as in its rhizosphere soil.

*The compound highlighted in blue was only found in the roots inoculated with GLAU-BT2.

**Supplementary references**

Ahmad, K., Thomas, N. F., Hadi, A. H. A., Mukhtar, M. R., Mohamad, K., Nafiah, M. A., Takeya, K., Morita, H., Litaudon, M., & Arai, H. (2010). Oppositinines A and B: new vasorelaxant β-carboline alkaloids from Neisosperma oppositifolia. *Chemical and Pharmaceutical Bulletin*, *58*(8), 1085–1087.

Ahmed, N. A., & Othman, A. S. (2024). Green fabrication of ZnO nanoparticles via spirulina platensis and its efficiency against biofilm forming pathogens. *Microbial Cell Factories*, *23*(1), 92.

AlAmery, S. F. (2020). Phytochemical profile and antifungal activity of stems and leaves methanol extract from the Juncus maritimus Linn. Juncaceae family against some dermatophytes fungi. *AIP Conference Proceedings*, *2290*(1).

Alexander, R., Kerby, A., Aubdool, A. A., Power, A. R., Grover, S., Gentry, C., & Grant, A. D. (2013). 4α‐phorbol 12, 13‐didecanoate activates cultured mouse dorsal root ganglia neurons independently of TRPV4. *British Journal of Pharmacology*, *168*(3), 761–772.

Alkon, D. L. (2022). *Treatment of amyotrophic lateral sclerosis using pkc activators*. Google Patents.

Altameme, H. J. (2017). A Chemical composition of Halophyte plant Frankenia pulverulenta L.(Frankeniaceae) in Iraq depending on GC-MS and FT-IR techniques. *J. Chem. Pharm. Sci*, *10*(1), 26–33.

Altameme, H. J., Hameed, I. H., & Kareem, M. A. (2015). Analysis of alkaloid phytochemical compounds in the ethanolic extract of Datura stramonium and evaluation of antimicrobial activity. *African Journal of Biotechnology*, *14*(19), 1668.

Barkatullah, Shabana, & Nafees, M. (2024). Chromatographic analysis and antioxidant potency of the crude extract of Xanthium spinosum in various fractions. *Biomedical Chromatography*, *38*(2), e5776.

Bhat, A. A., Gupta, G., Afzal, M., Thapa, R., Ali, H., Alqahtani, S. M., almalki, W. H., Kazmi, I., Alzarea, S. I., & Saleem, S. (2024). Polyphenol-loaded nano-carriers for breast cancer therapy: a comprehensive review. *BioNanoScience*, 1–19.

Boscaro, C., Schmidt, G., Cignarella, A., Dal Maso, L., Bolego, C., & Trevisi, L. (2024). The antiangiogenic effect of digitoxin is dependent on a ROS-elicited RhoA/ROCK pathway activation. *Biochemical Pharmacology*, *222*, 116049.

Chandrasekar, T., Rao, M. R. K., Kumar, R. V., Prabhu, K., Kumar, S. N., & Divya, D. (2015). *GC-MS analysis, antimicrobial, antioxidant activity of an Ayurvedic medicine, Nimbapatradi choornam.*

de Oliveira, J. R., Pereira, A. B. M., de Souza, H. I., Dos Santos, W. M., de Assunção, T. S. F., de Vito, F. B., de Souza, H. M., da Silva, P. R., da Silva, M. V., & Junior, V. R. (2024). Anti-inflammatory actions of aspirin-triggered resolvin D1 (AT-RvD1) in bronchial epithelial cells stimulated by cigarette smoke extract. *Prostaglandins & Other Lipid Mediators*, *172*, 106833.

Do, N. D., Ohbayashi, K., Naka, H., Nakada, K., & Ando, T. (2011). Identification and field evaluation of sex pheromone components of the pear barkminer moth, Spulerina astaurota. *Journal of Chemical Ecology*, *37*, 1222–1230.

Dong, W., Ni, Y., & Kokot, S. (2015). Differentiation of Mint (Mentha haplocalyx Briq.) from different regions in China using gas and liquid chromatography. *Journal of Separation Science*, *38*(3), 402–409.

Ediriweera, M. K., Cuong, D. M., & Cho, S. K. (2024). Cow milk derived-fat inhibits the proliferation of liver cancer-cells. *Applied Biological Chemistry*, *67*(1), 7.

El-Sheekh, M. M., Mousa, A. S. H., & Farghl, A. A. M. (2020). Biological control of Fusarium wilt disease of tomato plants using seaweed extracts. *Arabian Journal for Science and Engineering*, *45*, 4557–4570.

Fu, G.-M., Wang, Y.-H., Gao, S., Tang, M.-J., & Yu, S.-S. (2005). Five new cytotoxic triterpenoid saponins from the roots of Symplocos chinensis. *Planta Medica*, *71*(07), 666–672.

Gadhoumi, H., Dhouafli, Z., Yeddes, W., Serairi Beji, R., Miled, K., Trifi, M., Chirchi, A., Saidani Tounsi, M., & Hayouni, E. A. (2024). Biochemical Composition, Antioxidant Capacity and Protective Effects of Three Fermented Plants Beverages on Hepatotoxicity and Nephrotoxicity Induced by Carbon Tetrachloride in Mice. *Indian Journal of Microbiology*, *64*(1), 229–243.

Gamboa-Becerra, R., Desgarennes, D., Molina-Torres, J., Ramírez-Chávez, E., Kiel-Martínez, A. L., Carrión, G., & Ortiz-Castro, R. (2022). Plant growth-promoting and non-promoting rhizobacteria from avocado trees differentially emit volatiles that influence growth of Arabidopsis thaliana. *Protoplasma*, 1–20.

Guzzo, P. R., Manning, D. D., & Earley, W. (2015). *5-HT3 receptor modulators, methods of making, and use thereof*. Google Patents.

Hagström, Å. K., Wang, H.-L., Liénard, M. A., Lassance, J.-M., Johansson, T., & Löfstedt, C. (2013). A moth pheromone brewery: production of (Z)-11-hexadecenol by heterologous co-expression of two biosynthetic genes from a noctuid moth in a yeast cell factory. *Microbial Cell Factories*, *12*, 1–11.

Hashem, A. H., Shehabeldine, A. M., Abdelaziz, A. M., Amin, B. H., & Sharaf, M. H. (2022). Antifungal activity of endophytic Aspergillus terreus extract against some fungi causing mucormycosis: ultrastructural study. *Applied Biochemistry and Biotechnology*, *194*(8), 3468–3482.

He, J.-Y., Ma, N., Zhu, S., Komatsu, K., Li, Z.-Y., & Fu, W.-M. (2015). The genus Codonopsis (Campanulaceae): a review of phytochemistry, bioactivity and quality control. *Journal of Natural Medicines*, *69*, 1–21.

Hota, R. N., Nanda, B. K., Behera, B. R., & Bose, A. (2022). Ameliorative effect of ethanolic extract of Limnophila rugosa (Scrophulariaceae) in paracetamol-and carbon tetrachloride-induced hepatotoxicity in rats. *Future Journal of Pharmaceutical Sciences*, *8*(1), 6.

Hu, P. C., & Chen, B. H. (2002). Effects of riboflavin and fatty acid methyl esters on cholesterol oxidation during illumination. *Journal of Agricultural and Food Chemistry*, *50*(12), 3572–3578.

Hussein, H. M., Ubaid, J. M., & Hameed, I. H. (2016). Inscticidal activity of methanolic seeds extract of Ricinus communis on adult of callosobruchus maculatus (coleopteran: brauchidae) and analysis of its phytochemical composition. *International Journal of Pharmacognosy and Phytochemical Research*, *8*(8), 1385–1397.

Idan, S. A., Al-Marzoqi, A. H., & Hameed, I. H. (2015). Spectral analysis and anti-bacterial activity of methanolic fruit extract of Citrullus colocynthis using gas chromatography-mass spectrometry. *African Journal of Biotechnology*, *14*(46), 3131–3158.

Ishihara, H., Fujihara, H., Takada, Y., & Terazaki, H. (2005). *Fragrance composition*. Google Patents.

Johnson, T. O., Odoh, K. D., Nwonuma, C. O., Akinsanmi, A. O., & Adegboyega, A. E. (2020). Biochemical evaluation and molecular docking assessment of the anti-inflammatory potential of Phyllanthus nivosus leaf against ulcerative colitis. *Heliyon*, *6*(5).

Kassam, R., Yadav, J., Chawla, G., Kundu, A., Hada, A., Jaiswal, N., Bollinedi, H., Kamil, D., Devi, P., & Rao, U. (2021). Identification, characterization, and evaluation of nematophagous fungal species of Arthrobotrys and Tolypocladium for the management of Meloidogyne incognita. *Frontiers in Microbiology*, *12*, 790223.

Kolho, K.-L., Pessia, A., Jaakkola, T., de Vos, W. M., & Velagapudi, V. (2017). Faecal and serum metabolomics in paediatric inflammatory bowel disease. *Journal of Crohn’s and Colitis*, *11*(3), 321–334.

Kühn, H., Hayess, K., Holzhütter, H. G., Zabolotzski, D. A., Myagkova, G. I., & Schewe, T. (1991). Inactivation of 15-lipoxygenases by acetylenic fatty acids. *Biomedica Biochimica Acta*, *50*(7), 835–839.

Li, M., Han, G., Chen, H., Yu, J., & Zhang, Y. (2012). Chemical compounds and antimicrobial activity of volatile oils from bast and fibers of Apocynum venetum. *Fibers and Polymers*, *13*, 322–328.

Liang, Y. (2015). *Composition for flexible substrate and flexible substrate*. Google Patents.

Liu, B., Kaurilind, E., Zhang, L., Okereke, C. N., Remmel, T., & Niinemets, Ü. (2022). Improved plant heat shock resistance is introduced differently by heat and insect infestation: the role of volatile emission traits. *Oecologia*, *199*(1), 53–68.

Malathi, K., Anbarasu, A., & Ramaiah, S. (2017). Ethyl iso-allocholate from a medicinal rice Karungkavuni inhibits dihydropteroate synthase in Escherichia coli: A molecular docking and dynamics study. *Indian J Pharm Sci*, *78*(6), 780–788.

Maurya, A. (2020). Homogeneous catalytic oxidation of alkenes employing mononuclear vanadium complex with hydrogen peroxide. *Journal of the Iranian Chemical Society*, *17*, 3261–3269.

Mohy El-Din, S. M., & Mohyeldin, M. M. (2018). Component analysis and antifungal activity of the compounds extracted from four brown seaweeds with different solvents at different seasons. *Journal of Ocean University of China*, *17*, 1178–1188.

Mullaivendhan, J., Akbar, I., Ahamed, A., Gatasheh, M. K., Hatamleh, A. A., Raman, G., Manilal, A., & Kuzhunellil Raghavanpillai, S. (2024). Synthesis of a New Series of Anthraquinone-Linked Cyclopentanone Derivatives: Investigating the Antioxidant, Antibacterial, Cytotoxic and Tyrosinase Inhibitory Activities of the Mushroom Tyrosinase Enzyme Using Molecular Docking. *Drug Design, Development and Therapy*, 597–612.

Nabih, I. M. (1976). *New anti-malarial agents*. Google Patents.

Nand, P., Drabu, S., & Gupta, R. K. (2011). Antimicrobial investigation of Linum usitatissimum for the treatment of acne. *Natural Product Communications*, *6*(11), 1934578X1100601133.

Nes, W. D. (2011). Biosynthesis of cholesterol and other sterols. *Chemical Reviews*, *111*(10), 6423–6451.

Nowak, I., Issayeva, A., Dąbrowska, M., Wawrzyńczak, A., Jeleń, H., Łęska, B., Abubakirova, A., & Tleukeyeva, A. (2022). Possibilities of using medicinal plant extracts and salt-containing raw materials from the Aral region for cosmetic purposes. *Molecules*, *27*(16), 5122.

Özdemir, O., Yılmaz, N., Gok, M., & Kaya, M. O. (2022). Determination of antimicrobial and antioxidant activities of Lavandula angustifolia volatile oil. *Türkiye Tarımsal Araştırmalar Dergisi*, *9*(3), 265–273.

Palani, S., Raja, S., Kumar, R. P., Selvaraj, R., & Kumar, B. S. (2011). Evaluation of phytoconstituents and anti-nephrotoxic and antioxidant activities of Monochoria vaginalis. *Pakistan Journal of Pharmaceutical Sciences*, *24*(3).

Parveen, S., Akhtar, N., E-kobon, T., Burchmore, R., Hussain, A. I., & Akhtar, K. (2024). Biodesulfurization of organosulfur compounds by a trehalose biosurfactant producing Gordonia sp. isolated from crude oil contaminated soil. *World Journal of Microbiology and Biotechnology*, *40*(3), 103.

Pinnavaia, T. J. (1987). Swelling clays and related complex layered oxides. In *Chemical Physics of intercalation* (pp. 233–252). Springer.

PL, K. (1989). Aqueous humor dynamics: how PGF2α lowers intraocular pressure. *The Ocular Effect of Prostaglandins and Other Eicosanoids*.

Prabakaran, S., Ramu, L., Veerappan, S., Pemiah, B., & Kannappan, N. (2017). Effect of different solvents on volatile and non-volatile constituents of red bell pepper (Capsicum annuum L.) and their in vitro antioxidant activity. *Journal of Food Measurement and Characterization*, *11*, 1531–1541.

Ragunath, C., & Ramasubramanian, V. (2022). Dietary effect of Padina boergesenii on growth, immune response, and disease resistance against Pseudomonas aeruginosa in Cirrhinus mrigala. *Applied Biochemistry and Biotechnology*, *194*(5), 1881–1897.

Rajput, M., Bithel, N., & Vijayakumar, S. (2021). Antimicrobial, antibiofilm, antioxidant, anticancer, and phytochemical composition of the seed extract of Pongamia pinnata. *Archives of Microbiology*, *203*(7), 4005–4024.

Ratheesh, M., Sunil, S., Sheethal, S., Jose, S. P., Sandya, S., Ghosh, O. S. N., Rajan, S., Jagmag, T., & Tilwani, J. (2022). Anti-inflammatory and anti-COVID-19 effect of a novel polyherbal formulation (Imusil) via modulating oxidative stress, inflammatory mediators and cytokine storm. *Inflammopharmacology*, *30*(1), 173–184.

Saha, S. S., Dasgupta, P., Sengupta, S., & Ghosh, M. (2012). Synergistic effect of conjugated linolenic acid isomers against induced oxidative stress, inflammation and erythrocyte membrane disintegrity in rat model. *Biochimica et Biophysica Acta (BBA)-General Subjects*, *1820*(12), 1951–1970.

Savoia, D., Balestri, D., Grilli, S., & Monari, M. (2014). anti‐Dioxylation of Cyclohex‐4‐ene‐1, 2‐diamine Derivatives: Asymmetric Routes to Hydroxy‐and Amino‐Substituted Cyclohexane and 7‐Azanorbornane. *European Journal of Organic Chemistry*, *2014*(9), 1907–1914.

Sayed, H. M., Ramadan, M. A., Salem, H. H., Ahmad, I., Patel, H., & Fayed, M. A. A. (2023). Phytochemical Investigation, In silico/In vivo Analgesic, and Anti-inflammatory Assessment of the Egyptian Cassia occidentalis L. *Steroids*, *196*, 109245.

Shareef, H. K., Muhammed, H. J., Hussein, H. M., & Hameed, I. H. (2016). Antibacterial effect of ginger (Zingiber officinale) roscoe and bioactive chemical analysis using gas chromatography mass spectrum. *Oriental Journal of Chemistry*, *32*(2), 20–40.

Shibnev, V. A., Deryabin, P. G., Garaev, T. M., Finogenova, M. P., Botikov, A. G., & Mishin, D. V. (2017). Peptide carbocyclic derivatives as inhibitors of the viroporin function of RNA-containing viruses. *Russian Journal of Bioorganic Chemistry*, *43*, 517–525.

Singh, S., Maurya, A. K., Meena, A., Mishra, N., & Luqman, S. (2023). Myricitrin from bayberry as a potential inhibitor of cathepsin-D: prospects for squamous lung carcinoma prevention. *Food and Chemical Toxicology*, *179*, 113988.

Stansbury, J. W., Bowman, C. N., & Trujillo, M. (2014). *Dimer acid-derived dimethacrylates and use in dental restorative compositions*. Google Patents.

Tsai, J.-Y., Xia, C., Lin, C., Palacios, A. U., Oñate, E., Esteruelas, M. A., & Boudreault, P.-L. T. (2017). *Organic electroluminescent materials and devices*. Google Patents.

Udayaprakash, N. K., Ranjithkumar, M., Deepa, S., Sripriya, N., Al-Arfaj, A. A., & Bhuvaneswari, S. (2015). Antioxidant, free radical scavenging and GC–MS composition of Cinnamomum iners Reinw. ex Blume. *Industrial Crops and Products*, *69*, 175–179.

Varadharajan, R., Rajalingam, D., & Palani, S. (2016). GCMS/MS analysis and cardioprotective potential of Cucumis callosus on doxorubicin induced cardiotoxicity in rats. *Int. J. Pharm. Pharm. Sci*, *8*(9), 239–245.

Wang, N., Yao, X., Ishii, R., & Kitanaka, S. (2001). Antiallergic agents from natural sources. 3. Structures and inhibitory effects on nitric oxide production and histamine release of five novel polyacetylene glucosides from Bidens parviflora WILLD. *Chemical and Pharmaceutical Bulletin*, *49*(8), 938–942.

Winnett, V., Sirdaarta, J., White, A., Clarke, F. M., & Cock, I. E. (2017). Inhibition of Klebsiella pneumoniae growth by selected Australian plants: natural approaches for the prevention and management of ankylosing spondylitis. *Inflammopharmacology*, *25*, 223–235.

Yahaya, M. F., Osemeahon, S. A., Shagal, M. H., Maitera, O. N., Dass, P. M., & Yelwa, J. M. (2021). Antimicrobial, antioxidant, cytotoxicity profiles and chemical compositions of ethanolic extracts of Ficus polita and Ficus thonningii plant. *Journal of Research in Chemistry*, *2*, 4–10.

Yang, A., Liu, S., Zhang, Y., Chen, J., Fan, Y., Wang, F., Zou, Y., Feng, S., Wu, J., & Hu, Q. (2024). Regulation of RAS palmitoyltransferases by accessory proteins and palmitoylation. *Nature Structural & Molecular Biology*, *31*(3), 436–446.

Yoshitomi, T., Futashima, R., & Uda, T. (2021). *Bioelectrode and manufacturing method of bioelectrode*. Google Patents.
